# Gallbladder agenesis: An accidental finding during laparotomy for hepatic tumor

**DOI:** 10.1016/j.ijscr.2021.105875

**Published:** 2021-04-07

**Authors:** Ayad Ahmad Mohammed, Sardar Hassan Arif

**Affiliations:** Department of Surgery, College of Medicine, University of Duhok, Kurdistan Region, Iraq

**Keywords:** Biliary anomalies, Gall bladder agenesis, MRCP, Laparoscopy, Congenital anomalies

## Abstract

**Background:**

Anomalies of the biliary system are frequently encountered. Agenesis of the gall bladder is a rare. The exact incidence is not known as most cases are asymptomatic and diagnosed incidentally during surgeries for unrelated conditions or at autopsy. This anomaly may occur alone or in association with other anomalies such as other biliary anomalies, portal vein anomalies and other vascular anomalies, or in some cases hepatic, gastrointestinal or anomalies in other body systems.

**Case presentation:**

An elderly man diagnoses with segment VI liver tumor and planned for surgical resection. During surgery we accidentally discovered gall bladder agenesis with slightly dilated common bile duct. The tumor resected successfully and the final diagnosis was well differentiated with hepatocellular carcinoma.

**Conclusion:**

Careful search for the gall bladder must be done before diagnosing agenesis of the gall bladder. No conversion is required provided adequate visualization and search for the gall bladder is done during laparoscopy. Postoperative MCRP will define the biliary anatomy and to help the surgeons to have postoperative accurate decision.

## Highlights

• Anomalies of the biliary system are frequently encountered.
• Agenesis of the gall bladder is a rare.
• It may occur alone or in association with other anomalies.
• Careful search for the gall bladder must be done before diagnosing agenesis of the gall bladder.

## 1 Introduction

Anomalies of the biliary system are frequently encountered during medical practice. Agenesis of the gall bladder is a rare anatomical anomaly of the biliary system, in which there a complete absence of the gall bladder and or cystic duct. The exact incidence is not known as the majority of cases are asymptomatic and many cases are diagnosed incidentally during surgeries for unrelated conditions or at autopsy. It is reported in some literature that the incidence range between 0.01 % and 0.075 % [[1], [2], [3]].

This anomaly may occur alone or in association with other anomalies such as other biliary anomalies, portal vein anomalies and other vascular anomalies, or in some cases hepatic, gastrointestinal or anomalies in other body systems [2].

Most cases are asymptomatic provided it is not associated with other anomalies, patients may complain from biliary symptoms for long time before being diagnosed either by radiology or more commonly during surgery. Right upper quadrant abdominal pain is the commonest presentation, other symptoms may include nausea and vomiting, fatty food intolerance, dyspepsia and attacks of jaundice [2].

Most cases are missed during ultrasound examination which require high index of suspicion and great experience for the diagnosis. Hepato-biliary scintigraphy, cholangiography, CT-scan of the abdomen, endoscopic retrograde cholangio-pancreatography (ERCP), or magnetic resonance cholangio-pancreatography (MRCP) are greatly helpful and diagnostic, they show details of the anatomical structures and possibly show some of the associated congenital anomalies when present [2].

If the diagnosis is made during surgery, it is important to confirm the condition and exclude the possibility of ectopic gall bladder, most patients reported improvement after surgery which have been explained to be attributed to release of adhesions in this region and reduction of the pain [2].

The work of this report case has been reported in line with the SCARE 2018 criteria [4].

## 2 Patient information

A 77-year-old man presented with dull aching right hypochondrial pain for 1 month duration. The pain was poorly localized and radiated to the back. The patient had no history of jaundice, fever, nausea, or vomiting. There was no history of reported weight loss.

The patient had no history of chronic medical conditions. The past surgical history was negative.

### 2.1 Diagnostic assessment

Ultrasound of the liver revealed an evidence of 55*50 mm well defined iso-echoic mass with increased peripheral vascularity within the right lobe of the liver.

**Fig. 1:**
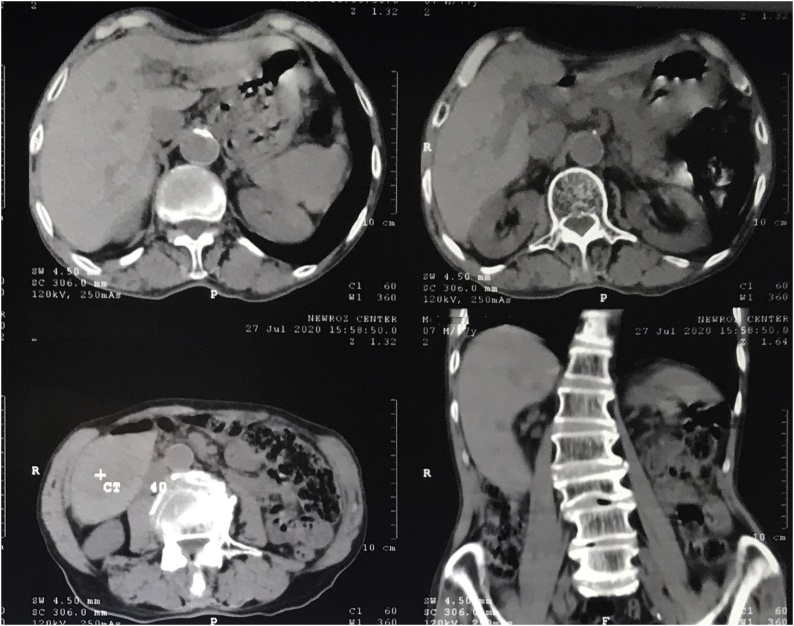
Native CT-scan of the abdomen showing liver tumor in segment VI, the gall bladder is not visualized in the films.

**Fig. 2:**
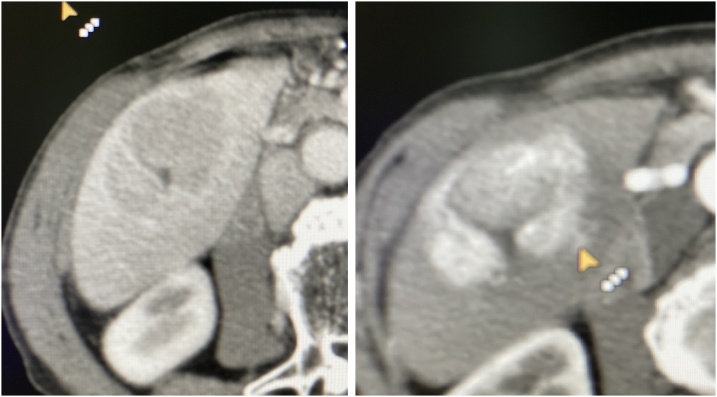
Contrast enhanced CT-scan of the abdomen showing liver tumor in segment VI with peripheral enhancement and rapid washout.

CT-scan of the abdomen showed hypo dense soft tissue mass within segment VI of the liver. After intravenous contrast injection the mass showed vivid enhancement with rapid washout, hypo-attenuation was noted in the portal phase with visualization of a feeding vessel to the tumor, the CT-scan findings were suggestive for hepatocellular carcinoma. Fig. 1, Fig. 2.

The hemoglobin level was normal (13.8 g/dl), the WBC were normal (10,500*10*^9^/L), and the platelets were normal (225*10*^9^/L). The blood sugar and the renal function test were normal. The serum levels of alpha-feto protein was normal (3.4 ng/mL), CA 19.9 was normal (20 U/mL), carcino-embryonic antigen was normal (4.62 ng/mL) and the CA 125 level was also normal (5.52 U/mL). The bleeding time and the coagulation profiles were normal.

### 2.2 Therapeutic intervention

**Fig. 3:**
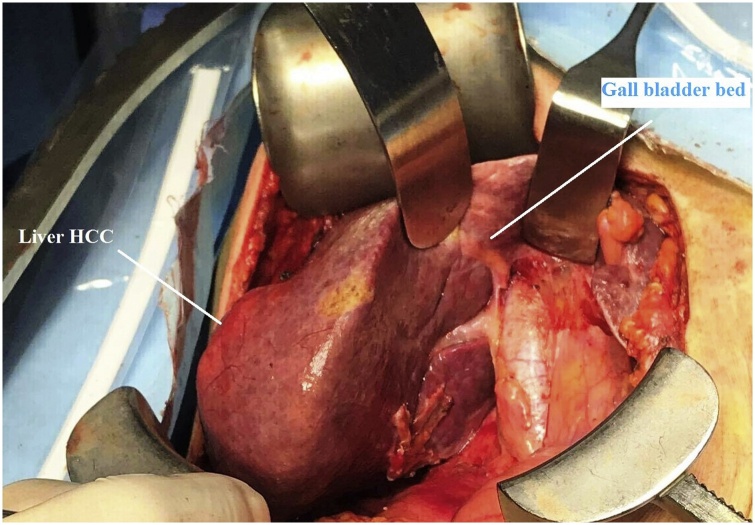
An intraoperative picture showing the liver tumor and the absence of the gall bladder.

An extended right subcostal incision was done and exploration of the peritoneal cavity was done, there was evidence of 5*5 cm mass related to liver segment VI. During surgery we accidentally discovered gall bladder agenesis with slightly dilated common bile duct. Fig. 3.

The tumor resected using radiofrequency device and sent for histopathological study.

**Fig. 4:**
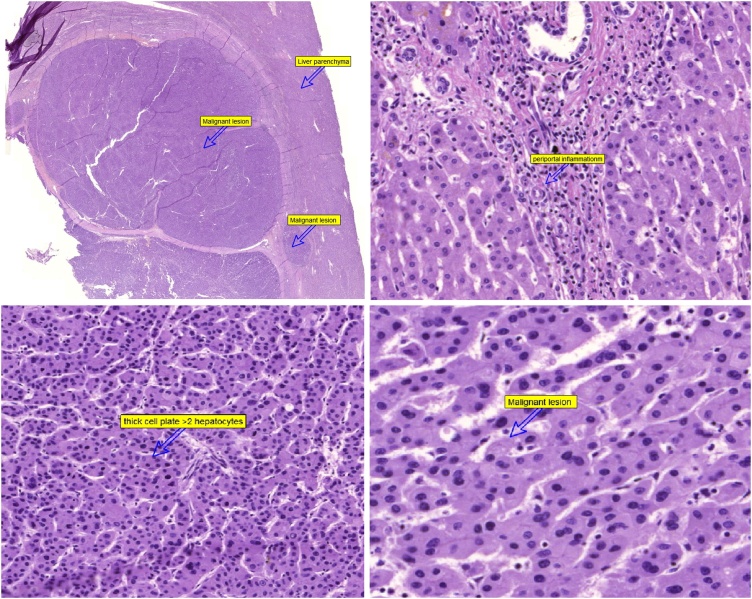
The histopathological appearance of the hepatic tumor.

The histopathological examination showed well differentiated proliferating hepatocytes, with formation of thick trabeculae and low grade atypia. The cells were negative for B-catenin, Glypecan-3, and CD10. The proliferative index was 35 %. The diagnosis was consistent with well differentiated with hepatocellular carcinoma. Fig. 4.

### 2.3 Follow-up and outcomes

The patient was admitted to the hospital for 5 days and discharged with no post-operative complications.

## 3 Discussion

Isolated gall bladder anomaly without agenesis of the extra-hepatic biliary system is usually diagnoses in adulthood because it is an asymptomatic condition, while when there agenesis of the extra-hepatic biliary system it is evident in the very early neonatal life. The condition is very rare and less than 500 cases are reported in literature [2].

The exact etiology is not known till now, most cases are often sporadic. Some case series are reported to affect many member of the same family suggesting that a genetic predisposition may play a role [5].

The main concern is that when gall bladder agenesis is discovered during surgery, in such condition the operating surgeon should prove that is a true agenesis rather than an ectopic embedded one by thorough examination of the retro-hepatic space, the falciform ligament, the lesser omentum, and the left side for possibility of left sided one [2,6,7].

The complain of the patients may be due to stones in the common bile duct, attacks of cholangitis, or dysfunction of the sphincter of Oddi. When there is no improvement after surgery, search for other possible causes must be done such as peptic ulcer disease, musculo-skeletal pain, or pain from other pathological sources [2,8,9].

There is a great debate about whether to convert the surgery from laparoscopic cholecystectomy to the conventional open technique when there is failure of gall bladder visualization. Most authors agree that no conversion is required provided adequate visualization and search for the gall bladder was done. Conversion to open surgery unduly increase the operation time and may result in increased morbidity with unnecessary prolongation of the hospital stay, in such situation postoperative MCRP is mandatory to define the biliary anatomy and to help the surgeons to have a more accurate decision. In cases of isolated gall bladder agenesis with no cystic duct agenesis, intra-operative cholangiography through the cystic duct may be done which will appropriately define the anatomy [10,11].

## Declaration of Competing Interest

The author has no conflicts of interest to declare.

## Sources of funding

None.

## Ethical approval

Ethical approval has been exempted by my institution for reporting this case.

## Consent

An informed written consent was taken from the family for reporting the case and the accompanying images.

## Author’s contribution

Dr Ayad Ahmad Mohammed and Dr Sardar Hassan Arif contributed to the concept of reporting the case and the patient data recording.

Drafting the work, design, and revision done by Dr Ayad Ahmad Mohammed.

Final approval of the work to be published was done by Dr Ayad Ahmad Mohammed and Dr Sardar Hassan Arif.

## Registration of research studies

Not applicable.

## Guarantor

Dr Ayad Ahmad Mohammed is guarantor for the work.

## Provenance and peer review

Not commissioned, externally peer-reviewed.

## Patient’s perspective

I was really concerned about the liver tumor, as this was an accidental finding and I was symptomless until this age, I don’t think it will cause great problems.
